# Alcohol intake, wine consumption and the development of depression: the PREDIMED study

**DOI:** 10.1186/1741-7015-11-192

**Published:** 2013-08-30

**Authors:** Alfredo Gea, Juan J Beunza, Ramón Estruch, Almudena Sánchez-Villegas, Jordi Salas-Salvadó, Pilar Buil-Cosiales, Enrique Gómez-Gracia, María-Isabel Covas, Dolores Corella, Miquel Fiol, Fernando Arós, José Lapetra, Rosa-María Lamuela-Raventós, Julia Wärnberg, Xavier Pintó, Lluis Serra-Majem, Miguel A Martínez-González

**Affiliations:** 1 Department of Preventive Medicine and Public Health, Medical School-Clinica Universidad de Navarra, Pamplona, Spain; 2 School of Medicine, Universidad Europea de Madrid, Madrid, Spain; 3 CIBER Fisiopatología de la Obesidad y Nutrición (CIBERObn), Instituto de Salud Carlos III, Madrid, Spain; 4 Department of Internal Medicine, Hospital Clinic, University of Barcelona, Barcelona, Spain; 5 Department of Clinical Sciences, University of Las Palmas de Gran Canaria, Las Palmas de Gran Canaria, Spain; 6 Human Nutrition Unit, IISPV, Universitat Rovira i Virgili, Reus, Spain; 7 Spain Primary Care, Servicio Navarro de Salud, Osasunbidea, Pamplona, Spain; 8 Department of Preventive Medicine and Public Health, Faculty of Medicine, University of Málaga, Málaga, Spain; 9 Lipids and Cardiovascular Epidemiology Research Unit, Institut Municipal d’Investigació Mèdica (IMIM), Barcelona, Spain; 10 Department of Preventive Medicine, University of Valencia, Valencia, Spain; 11 Institute of Health Sciences (IUNICS), University of Balearic Islands, Palma de Mallorca, Spain; 12 Department of Cardiology, Araba University Hospital, Vitoria, Spain; 13 Department of Family Medicine, Primary Care Division of Sevilla, Centro de Salud San Pablo, Sevilla, Spain; 14 Nutrition and Food Science Department– XaRTA, INSA, University of Barcelona, Barcelona, Spain; 15 Internal Medicine Service, Hospital of Bellvitge, Hospitalet de Llobregat, Spain

**Keywords:** Wine, Alcohol, Depression, Cohort

## Abstract

**Background:**

Alcoholic beverages are widely consumed. Depression, the most prevalent mental disorder worldwide, has been related to alcohol intake. We aimed to prospectively assess the association between alcohol intake and incident depression using repeated measurements of alcohol intake.

**Methods:**

We followed-up 5,505 high-risk men and women (55 to 80 y) of the PREDIMED Trial for up to seven years. Participants were initially free of depression or a history of depression, and did not have any history of alcohol-related problems. A 137-item validated food frequency questionnaire administered by a dietician was repeated annually to assess alcohol intake. Participants were classified as incident cases of depression when they reported a new clinical diagnosis of depression, and/or initiated the use of antidepressant drugs. Cox regression analyses were fitted over 23,655 person-years.

**Results:**

Moderate alcohol intake within the range of 5 to 15 g/day was significantly associated with lower risk of incident depression (hazard ratio (HR) and 95% confidence interval (95% CI) = 0.72 (0.53 to 0.98) versus abstainers). Specifically, wine consumption in the range of two to seven drinks/week was significantly associated with lower rates of depression (HR (95% CI) = 0.68 (0.47 to 0.98)).

**Conclusions:**

Moderate consumption of wine may reduce the incidence of depression, while heavy drinkers seem to be at higher risk.

## Background

Most cultures and countries include alcoholic beverages as part of their usual diet. Alcohol intake is different over world regions regarding the habitual type of beverage and the pattern of consumption (frequency and average intake). In general terms, the consumption of alcoholic beverages is increasing worldwide [1]. Unipolar depression is the most prevalent mental disorder in the world and it is increasing steadily [2].

The simultaneous presence of alcohol-related problems and depression is common [3]. However, problematic intake needs to be distinguished from moderate intake. Problematic alcohol intake may be associated with depression not only because of increased intake of ethanol but also because of other alcohol-related unhealthy lifestyles or because of the social environment surrounding problematic drinkers (job loss, family problems, financial problems or other addictions). Any of these circumstances may be a potential trigger for depression, even in the absence of a specific detrimental role for ethanol.

Unipolar depression and cardiovascular disease are likely to share some common pathophysiological mechanisms. Moderate alcohol intake, especially alcohol from wine, has been repeatedly reported to be inversely associated with the incidence of cardiovascular disease [4-6]. Some of the responsible mechanisms for this inverse association are likely to be involved also in a reduced risk of depression [7,8]. In the existing literature about non-problematic alcohol use and depression, longitudinal studies are inconsistent [9-20]. However, none of them has investigated the specific role of each beverage. Neither have they used repeated measurements on alcohol intake during follow-up to update the information on exposure to alcohol.

We prospectively evaluated the incidence of depression among light to moderate drinkers from an older Mediterranean cohort at high cardiovascular risk using repeated measurements of intake. An interesting characteristic of this population is that wine was the most frequently consumed alcoholic beverage. We also evaluated specifically the association of wine with incident depression, using repeated measurements of wine consumption.

## Methods

### Subjects

We assessed participants from the PREDIMED Study (“Prevención con Dieta Mediterránea” (Prevention with Mediterranean Diet)), which is a large, parallel-group, randomized, multicenter, controlled, clinical trial conducted in Spain. The aim of the PREDIMED trial was to assess the effect of the Mediterranean diet on the primary prevention of cardiovascular disease (http://www.predimed.es; http://www.predimed.org) [21].

Participants were men and women aged 55 to 80 and 60 to 80 years, respectively. They were free of cardiovascular disease at baseline and met at least one of the two following criteria: type 2 diabetes mellitus or the presence of three or more coronary heart disease risk factors.

Previous history of cardiovascular disease, any severe chronic illness, history of food allergy, intolerance to olive oil or nuts, drug addiction or chronic alcoholism were exclusion criteria in the PREDIMED trial. Subjects were screened for problematic use of alcohol using the CAGE questionnaire (a four-item screening tool) [21] and they were not included in the trial if they answered positively to at least two questions on this questionnaire.

Participants who met the previous inclusion criteria (n = 7,447) were randomly assigned to three interventions: Mediterranean diet supplemented with extra virgin olive oil, Mediterranean diet supplemented with mixed nuts or a control group. They were annually interviewed by a dietician, obtaining information about lifestyle, diet and incident diseases. Further aspects of the methods and design of the PREDIMED trial have been reported elsewhere in detail [22,23]. The Research and Ethic Committee of the Hospital Clínic (Barcelona, Spain), accredited by the Department of Health and Human Services and regulated by the Federalwide Assurance for the Protection of Human Subjects of International (Non-US) Institutions # 00000738 approved the study protocol on 16 July 2002. This trial has been registered in the London Current Controlled Trials with the number ISRCTN 35739639 [24]. All participants provided written informed consent.

Out of 7,447 participants, we excluded 153 participants with total energy intake out of predefined limits (800 and 4,000 Kcal/day for men and 500 and 3,500 Kcal/day for women) [25]. Another 1,579 subjects who reported at baseline prevalent depression, previous history of depression or use of antidepressant drugs were also excluded (to ensure the temporal sequence and, therefore, avoid reverse causation bias). Additionally, 210 participants were lost to follow-up or did not have alcohol information and were also excluded. Finally, a subsample of 5,505 participants was included in the analyses of the present study (Additional file 1).

### Exposure assessment

Alcohol intake was assessed at baseline with a validated 137-item semi-quantitative food-frequency questionnaire (FFQ), which included nine questions on consumption of different alcoholic beverages (different types of wine, beer and spirits). In the validation study for the Spanish version of this questionnaire, the intra-class correlation coefficient between alcohol intake from the FFQ and repeated food records was 0.82 [26]. This FFQ was repeatedly administered each year during follow-up.

In order to update alcohol information, we considered alcohol intake using repeated measurements of diet from all available FFQs. Participants were divided into four groups according to their alcohol intake: abstainers, those who reported drinking less than 5 g/day, those with an intake ranging from >5 to 15 g/day, and the fourth group with an intake higher than 15 g/day.

To assess the specific role of wine, participants were divided into five groups according to their average number of weekly drinks of wine: abstainers, less than one drink/week, one to less than two drinks/week, two to seven drinks/week, and more than seven drinks/week. Participants who did not drink wine but drank other types of alcoholic beverages were excluded from these specific analyses.

### Outcome assessment

An incident case of depression was defined as a diagnosis of depression made by a physician and reported by participants in any of the follow-up interviews, or a positive report of habitual use of antidepressant drugs.

### Confounder assessment

We obtained information about medical, socio-demographic, anthropometric and lifestyle variables with standardized questionnaires (see http://www.predimed.es) at the baseline interview.

### Statistical analysis

Cox regression models were used to assess the relationship between categories of baseline alcohol intake and the subsequent incidence of depression during follow-up. Hazard ratios (HR) and their 95% confidence intervals (95% CI) were calculated using the abstainers group as the reference category. Entry time was defined as the date at recruitment. Exit time was defined as the date at diagnosis of depression for cases and, for participants who did not develop depression, as the date when completing the last interview, 1 December 2010, or date at death, whichever came first. We adjusted the multivariable models for the following potential confounders: age, sex, smoking status (non-smokers, ex-smokers and current smokers), physical activity (MET-min/d), total energy intake (Kcal/day), baseline body mass index (kg/m^2^), marital status (married or not), intervention group (Mediterranean diet + virgin olive oil, Mediterranean diet + nuts, low fat diet), recruiting center (14 centers), educational level (in five categories from illiterate to university graduate), and number of persons living at home (living alone or not). We evaluated the interaction between sex and alcohol intake on the development of depression by calculating the likelihood ratio test between the fully adjusted model and the same model adding the interaction product-term. To take advantage of the yearly repeated measurements of diet, we updated the calculation of alcohol intake for participants every year from baseline to the incidence of depression from the repeated FFQs. To update alcohol intake with all available longitudinal data, and to analyze the association between alcohol intake and depression at all time-points simultaneously, we used generalized estimating equations (GEE) with Stata 12.0 (StataCorp, College Station, TX, USA). We assumed a binomial distribution with logit models and the unstructured matrix as the working correlation structure [27]. We defined the cohort risk as participants who remained free of depression at the beginning of each two-year follow-up period. Participants who had been classified as incident cases were excluded from subsequent follow-up. Then, only the first incident case of depression was considered as outcome. In the analysis of repeated measurements, to allow for a sufficiently long follow-up period, we modeled the incidence of depression in relation to the cumulative average alcohol intake from all available dietary questionnaires up to the start of each two-year follow-up interval, but we excluded cases occurring in the first year of that period and took into account only new cases occurring after one year. For example, for a participant recruited in 2005 the incidence of depression from 2007 through 2008 was related to the alcohol intake reported on the 2005 and 2006 questionnaires. Therefore, we assumed, an induction period longer than one year but not longer than two years and alcohol intake was considered as the cumulative average of all available FFQs, from baseline to the beginning of each two-year follow-up period. Several sensitivity analyses were conducted refitting Cox regression analyses after a) establishing the 1^st^ and the 99^th^ or b) the 5^th^ and 95^th^ percentiles of energy intake as allowable limits, c) after excluding diabetics, d) after excluding participants older than 75 years, e) after excluding participants younger than 65 years, f) after depurating the abstainers’ group (excluding participants who reported any alcohol intake throughout their life but not currently at baseline), g) after including participants with a prior history of depression at baseline and including this variable in the multiple adjusted model, and h) after establishing a two- to three-year induction period (instead of one to two years) between exposure and outcome in the repeated measurement analyses. Moreover, to assess the influence of wine consumption on the development of depression, we conducted both Cox regression and GEE analyses, using abstainers as the reference category. In these analyses, we adjusted for the same potential confounders as mentioned above, and also for alcohol intake from other sources. Additionally, we re-ran the analyses for wine consumption among only-wine drinkers to control for confounding by other alcoholic beverages.

Moreover, we evaluated the potential non-linear association between alcohol intake and incident depression with the use of restricted cubic splines, for participants drinking no more than 80 grams of alcohol per day (99.42% of participants) [28].

Finally, we examined the incidence and recovery from depression applying fixed-effects regression as suggested by Fergusson *et al*. [29] to control for observed and non-observed factors that do not vary for a subject over time.

## Results

The main characteristics of the 5,505 participants (2,683 males and 2,822 females) according to their total alcohol intake are shown in Table 1. As mentioned before, this Mediterranean population consisted of older men and women, with a mean age of 67 years. Higher alcohol intake was positively associated with being male (88% of participants drinking more than 15 g/day were male), practicing more leisure-time physical activity, being a current or former smoker, having higher total energy intake, being married, but it was inversely associated with educational level.

**Table 1:** Baseline characteristics of participants according to baseline alcohol intake

	***Alcohol intake categories (g/day)***			
	**0**	**>0 to 5**	**>5 to 15**	**>15**
N	1,818	1,356	1,279	1,052
Sex (female %)	78	59	38	12
Age (years)	68.5 (6.1)	67.0 (6.1)	66.4 (6.2)	65.6 (6.1)
BMI (kg/m^2^)	30.3 (4.1)	30.0 (3.9)	29.4 (3.5)	29.3 (3.3)
Leisure time physical activity (MET-min/d)	201 (210)	230 (232)	267 (276)	318 (270)
Secondary school or higher (%)	13	23	28	36
Current smokers (%)	7	12	17	29
Former smokers (%)	15	24	36	44
Marital status (% married)	71	78	84	89
Living alone (%)	12	9	7	5
Total energy intake (Kcal/day)	2,054 (511)	2,184 (509)	2,330 (511)	2,595 (533)
Alcohol (g/day)	-	2.0 (1.4)	9.8 (2.7)	35 (17)
Wine (g alcohol/day)	-	1.3 (1.4)	7.4 (3.7)	25 (16)
Beer (g alcohol/day)	-	0.5 (0.9)	1.7 (2.7)	4.8 (8.4)
Spirits (g alcohol/day)	-	0.2 (0.5)	0.6 (1.5)	4.2 (8.8)

Mean and Standard Deviations, or %. The PREDIMED Study 2003 to 2010. BMI, body mass index; MET, metabolic equivalent task.

A total of 443 incident cases of depression were identified during the follow-up period, which accrued 23,655 person-years.

The Cox regression analysis using baseline alcohol intake as exposure showed an inverse association between total alcohol intake and the incidence of depression, with no significant linear or quadratic trend in the multiple-adjusted analyses. In the multiple-adjusted model, only the association for the category of low-to-moderate intake (>5 to 15 g/day) remained statistically significant (HR (95% CI) = 0.72 (0.53 to 0.98)) (Table 2). Examining the association for those participants who drank more than 40 g/day, we found that they were at higher risk but this association was not statistically significant, probably due to the small number of heavy drinkers (HR (95% CI = 1.34 (0.69 to 2.59)).

**Table 2:** Risks of depression according to categories of daily alcohol intake

***Alcohol intake categories (g/day)***	**0**	**>0 to 5**	**>5 to 15**	**>15**	***P *for liner trend**
	Baseline alcohol intake^a^				
Cases/Person-years	195/7,777	114/5,728	79/5,390	55/4,760	
Crude model	1 (Ref.)	0.79 (0.63 to 1.00)	0.59 (0.46 to 0.77)	0.44 (0.33 to 0.60)	<0.001
Age and sex-adjusted	1 (Ref.)	0.91 (0.72 to 1.15)	0.81 (0.62 to 1.07)	0.81 (0.58 to 1.14)	0.347
Multiple-adjusted model^c^	1 (Ref.)	0.97 (0.75 to 1.25)	0.72 (0.53 to 0.98)	0.79 (0.53 to 1.16)	0.522
	Updated alcohol intake^b^				
Crude model	1 (Ref.)	0.63 (0.49 to 0.81)	0.49 (0.37 to 0.66)	0.37 (0.26 to 0.52)	<0.001
Age and sex-adjusted	1 (Ref.)	0.73 (0.56 to 0.94)	0.71 (0.52 to 0.97)	0.71 (0.48 to 1.05)	0.727
Multiple-adjusted model^c^	1 (Ref.)	0.73 (0.57 to 0.95)	0.69 (0.50 to 0.96)	0.69 (0.46 to 1.04)	0.773

^a^Hazard ratios (95% confidence intervals) for incident depression according to categories of baseline daily alcohol intake.

^b^Relative risks (95% confidence intervals) for categories of updated alcohol intake, using repeated measurements of diet during follow-up. To avoid reverse causality bias, an induction period of at least one year, but no longer than two years was assumed. We considered as incident cases of depression those occurring only during the second year of every two-year follow-up interval.

^c^Adjusted for age, sex, smoking, physical activity (MET-min/d), total energy intake (Kcal/day), baseline body mass index (kg/m^2^), marital status, intervention group, recruiting center, educational level and the number of persons living at home.

The PREDIMED Study 2003 to 2010.

In the repeated-measurement analyses using as exposure the yearly updated information on alcohol intake, we observed a stronger inverse association. We found a statistically significant inverse association for light drinkers (>0 to 5 g/day) with a relative risk (95% CI) of 0.73 (0.57 to 0.95) and also for low-to-moderate drinkers (>5 to 15 g/day) with a relative risk of 0.69 (0.50 to 0.96). We found no effect modification of alcohol intake by sex (*P* = 0.45). Although the interaction was non-significant, we re-ran the analyses separately for men and women to take into account biological differences between the sexes. The results are available in the supplementary material (Additional file 2).

**Figure 1:**
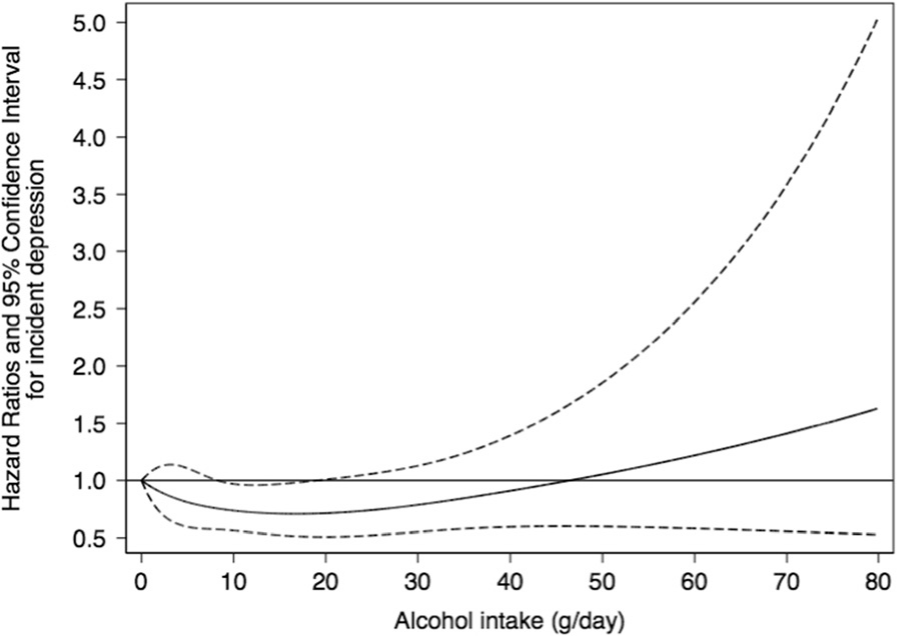
**Dose–response relationship between alcohol intake(g/day)and depression risk(HR and 95%CI).** Restricted cubic spline model. The PREDIMED Study 2003 to 2010. The black line represents the HR and the dashed lines represent the 95% confidence interval. Adjusted for age, smoking, physical activity (MET-min/d), total energy intake (Kcal/day), baseline body mass index (kg/m^2^), marital status, intervention group, recruiting center, educational level and the number of persons living at home.

To account for a non-linear association, we used restricted cubic spline analysis. The U-shaped association found, represented in Figure 1, showed a consistent protection against incident depression for baseline moderate alcohol intake.

Drinking wine (two to seven drinks/week) was inversely associated with the incidence of depression (Table 3). In the multiple-adjusted model, we found a HR (95% CI) of 0.68 (0.47 to 0.98) for the baseline consumption of two to seven drinks/week of wine. Using repeated measurements to update wine consumption, we also observed a strengthening of this inverse association with a relative risk of 0.57 (0.40 to 0.79) for those who drank two to seven drinks/week of wine. In our sample, 1,350 participants were only-wine drinkers. Among them the results were consistent with those previously discussed (Table 3). Wine consumption accounted for 82% of alcohol intake variability in this sample. The number of only-beer drinkers was just 308 and the number of only-spirits drinkers was 61. Only 1.63% of participants drank more than one drink/day of beer, and only 0.09% of spirits. Consequently, we could not do for beer or spirits the type of separate analysis that we did for wine. In order to investigate possible sources of bias in the estimation of the relationship between alcohol intake and depression, we conducted several sensitivity analyses (Table 4). Results did not substantially change except when excluding diabetics and for other analyses with a substantial number of exclusions, probably due to a lack of statistical power.

**Table 3:** Risks of depression according to weekly wine consumption

***Wine: drinks/week***	***Abstainers***	**<1**	**1 to<2**	**2 to 7**	**>7**
	Baseline wine consumption^a^				
Cases/Person-years	195/7,777	44/2,461	28/1,708	57/3,183	32/6,828
Age and sex-adjusted model	1 (Ref.)	1.05 (0.78 to 1.42)	0.82 (0.55 to 1.22)	0.75 (0.54 to 1.05)	0.79 (0.60 to 1.04)
Multiple-adjusted model^c^	1 (Ref.)	1.00 (0.72 to 1.39)	0.93 (0.61 to 1.43)	0.70 (0.48 to 1.00)	0.78 (0.57 to 1.06)
Multiple-adjusted model^d^	1 (Ref.)	0.99 (0.71 to 1.37)	0.92 (0.60 to 1.41)	0.68 (0.47 to 0.98)	0.76 (0.56 to 1.04)
	Updated wine consumption^b^				
Age-adjusted model	1 (Ref.)	0.64 (0.46 to 0.89)	0.86 (0.58 to 1.27)	0.59 (0.43 to 0.82)	0.70 (0.48 to 1.02)
Multiple-adjusted model^c^	1 (Ref.)	0.64 (0.46 to 0.89)	0.87 (0.58 to 1.29)	0.57 (0.41 to 0.80)	0.68 (0.46 to 1.01)
Multiple-adjusted model^d^	1 (Ref.)	0.63 (0.46 to 0.89)	0.86 (0.58 to 1.28)	0.57 (0.40 to 0.79)	0.66 (0.45 to 0.99)
	Baseline wine consumption (Only wine-drinkers)^a^				
Cases/Person-years	195/7,777	40/1,454	13/752	25/1,363	26/2,236
Age-adjusted model	1 (Ref.)	1.13 (0.80 to 1.59)	0.79 (0.45 to 1.38)	0.86 (0.56 to 1.30)	0.59 (0.39 to 0.90)
Multiple-adjusted model^c^	1 (Ref.)	1.18 (0.81 to 1.72)	0.91 (0.49 to 1.69)	0.81 (0.50 to 1.30)	0.55 (0.35 to 0.87)
	Updated wine consumption (Only wine-drinkers)^b^				
Age-adjusted model	1 (Ref.)	0.54 (0.34 to 0.87)	0.93 (0.52 to 1.66)	0.58 (0.36 to 0.92)	0.52 (0.24 to 1.14)
Multiple-adjusted model^c^	1 (Ref.)	0.55 (0.34 to 0.87)	0.93 (0.52 to 1.66)	0.57 (0.36 to 0.92)	0.52 (0.23 to 1.18)

^a^Hazard ratios (95% confidence intervals) for incident depression according to categories of baseline daily alcohol intake.

^b^Relative risks (95% confidence intervals) for categories of updated alcohol intake, using repeated measurements of diet during follow-up. To avoid reverse causality bias, an induction period of at least one year, but no longer than two years was assumed. We considered as incident cases of depression those occurring only during the second year of every two-year follow-up interval.

^c^Adjusted for age, smoking, physical activity (MET-min/d), total energy intake (Kcal/day), baseline body mass index (kg/m^2^), marital status, intervention group, recruiting center, educational level and the number of persons living at home.

^d^Adding alcohol from sources other than wine to the previous multiple-adjusted model.

The PREDIMED Study 2003 to 2010.

**Table 4:** Sensitivity analyses

	**Total cases/person-years**	**>5 to 5 g/day**
Overall	443/23,655	0.72 (0.53 to 0.98)
Energy limits: percentiles 5 to 95	403/21,681	0.78 (0.57 to 1.06)
Energy limits: percentiles 1 to 99	444/23,675	0.71 (0.53 to 0.96)
Excluding diabetics	227/11,653	0.96 (0.64 to 1.44)
Excluding participants older than 75	389/21,200	0.73 (0.56 to 1.25)
Excluding participants younger than 65	307/14,785	0.75 (0.52 to 1.06)
Depurating abstainers’ group^a^	422/22,882	0.75 (0.55 to 1.02)
Including participants with prior depression at baseline	817/25,868	0.72 (0.57 to 0.90)
Updated alcohol intake assuming induction period: 1 to 2 years^b^	-	0.69 (0.50 to 0.96)
Updated alcohol intake assuming induction period: 2 to 3 years^b^	-	0.68 (0.47 to 1.00)

^a^After excluding those participants who reported any alcohol intake throughout their life but not currently at baseline.

^b^Repeated-measurement analysis. Relative risks (95% confidence interval) for incident depression according to yearly updated measurements of alcohol intake. Considering incident cases during the last year of every two-year follow-up interval.

The PREDIMED Study 2003 to 2010.

All adjusted for age, sex, smoking, physical activity (MET-min/d), total energy intake (Kcal/day), baseline body mass index (kg/m^2^), marital status, intervention group, recruiting center, educational level, the number of persons living at home and alcohol intake at baseline.

Results were also consistent when we investigated the incidence and recovery from depression using fixed-effects models (Additional file 3).

## Discussion

We found that total low-to-moderate alcohol drinking (5 to 15 g/day) was associated with lower risk of depression. A stronger inverse association was found for low-to-moderate wine-drinkers (two to seven glasses/week).

These results seem paradoxical as previous studies found a direct association between alcohol in higher amounts, especially Alcohol Use Disorders (AUD), and depressive symptoms, [11,17-20,30]. However, lower amounts of alcohol intake might exert a protection as it has been observed for coronary heart disease (CHD). In fact, it is believed that depression and CHD share common pathophysiological mechanisms [30,31]. Only one previous cohort, including participants with a high educational level, assessed low-to-moderate levels of intake and found a similar inverse association (HR (95% CI); 0.65 (0.49 to 0.86)) for the same range of exposure 5 to 15 g/day that we have observed [9]. The results of our study and that previous cohort are, therefore, closely consistent. The practical absence of heavy drinkers in our sample and in that cohort, together with the large proportion of participants exposed to low-to-moderate intakes are likely explanations for the diverging results with respect to cohorts including heavy drinkers. The results of those studies have to be carefully interpreted. In several previous cross-sectional studies, a strong positive association was found. Therefore, the definition of a clear temporal sequence is necessary to preclude the possibility of a reverse causation bias [3]. Some longitudinal studies have assessed the reverse direction of this association, that is, the consequences of depression on alcohol intake. They have found direct associations; subjects with depression tend to increase their alcohol intake as a consequence of their mood disorder [2]. Therefore, in some cross-sectional analyses, the positive association found is likely to be the result of the direct effect of depression on alcohol intake, which may overcome the possible inverse association between low-to-moderate alcohol intake and depression risk. They can also capture the detrimental effects of higher amounts of alcohol intake on depression risk. In contrast, in a longitudinal analysis, the temporal sequence is preserved, and reverse causation bias is less likely. In order to minimize the risk of that bias, we assumed an induction period of at least one year and we conducted a sensitivity analysis assuming an alternative induction period of two to three years in the repeated measurement analyses. Early cases may be subclinical cases of depression at the beginning of the interval and may correspond to subjects who changed their alcohol intake before developing overt clinical depression. The fact that the results of these sensitivity analyses are consistent with the main results gives further support to our findings. Moreover, incident cases of depression did not change their alcohol intake differently from those who were not incident cases (*P* >0.1; data not shown).

The differences between our results and those of previous studies could also be explained by the different average alcohol intake between populations, the different pattern of consumption and especially because the predominant type of beverage consumed is different, wine being the preferential beverage in our cohort. The PREDIMED trial includes an older, traditional Spanish Mediterranean population, that consumed chiefly wine, and mainly in a context of socialization with family or friends. Subjects with problematic use of alcohol (≥2 positive answers in the CAGE questionnaire) [21] were explicitly excluded from this trial because this was one of the exclusion criteria. Moreover, they were in a trial where an intervention with a Mediterranean diet was done, including the advice to consume wine moderately only for those who were already wine drinkers at inception. However, the intervention designed for this trial did not attain any significant change in average levels of alcohol intake (−1.4 g/day, −1.4 g/day, and −0.9 g/day in the Mediterranean diet + virgin olive oil, Mediterranean diet + nuts and control groups, respectively) without any between-group significant difference. In addition, the Mediterranean diet has also been associated with lower incidence of depression [32]. Therefore, dummy variables for the two active intervention groups were included in the multiple-adjusted model as potential confounders.

The relationship between alcohol intake and depression is biologically different among men and women [20,33,34]. For instance, alcohol bioavailability is different between sexes [35], and women seem to be more likely to develop depression [36]. Therefore, we presented the analyses stratified by sex despite there being no evidence of statistical interaction by sex.

Besides the sensitivity analysis mentioned above, we conducted eight other sensitivity analyses to explore further potential sources of bias. The reference category in the main analysis was the abstainers’ group. This group may include former drinkers. Therefore, we excluded from the abstainers’ group all participants who reported drinking alcohol ever in her/his past life, and the results remained similar. However, the results became non-significant after the exclusion of prevalent diabetics at baseline. This fact may be explained by a loss of statistical power because almost 50% of our participants were diabetics. Alternatively, it might be attributed to a better disease classification among diabetic than among non-diabetic participants. Diabetics usually are more likely to have more frequent medical visits due to their diabetes; therefore, they have more opportunities to receive a medical diagnosis of depression than non-diabetic patients.

To our knowledge, with the exception of the recent results of the University of Navarra follow-up Study (“Seguimiento Universidad de Navarra” (SUN cohort)), no other large prospective cohort study has previously reported conclusive results about the relationship between specific alcoholic beverages and incident depression. Non-alcoholic components of wine can account for the inverse association found in our study. For instance, trans-resveratrol has been postulated as a neuroprotective substance [37-40]. Previous investigations suggest that the hippocampal complex may play a role in the development of major depression [41]. This neuroprotection applied to the hippocampus may prevent moderate wine drinkers from developing depression.

An alternative potential explanation for this association can be that wine-drinkers or moderate drinkers might be healthier in other aspects than non-wine drinkers or non-moderate drinkers, as it has been suggested for other outcomes [42]. In order to account for confounding by these factors, several lifestyle variables, including quality of the diet and several indicators of an overall healthy lifestyle and health consciousness, had been included in the multiple-adjusted model.

Some important strengths of the present study are its prospective design, the use of repeated measurements of alcohol intake during follow-up, the large sample size of a very homogeneous population and the good adjustment for potential confounders. In addition, the high prevalence of low-to-moderate average alcohol intake with almost null prevalence of excessive drinking together with the preference for wine consumption offered a unique opportunity to investigate the influence of these two particular factors (light-to-moderate drinking and wine consumption), while it limited our power to detect associations between heavy drinking and depression.

Inherent to nutritional epidemiology methods, we have to point out the possibility of some degree of misclassification in the dietary assessment methods. However, we used a FFQ extensively validated in Spain for the dietary assessment [26,43,44]. In addition, the correlation coefficient in the validation study was higher for alcohol than for most other nutrients. Moreover, if there is some misclassification it will be more likely non-differential and, therefore, the association would probably be driven towards the null value. Another limitation in our study is that we are not exclusively using a clinical diagnosis of depression. Probably we are achieving a high specificity at the expense of losing sensitivity. Moreover, there is a possibility that patterns of alcohol consumption may be associated with decisions to seek care. If heavy drinkers were less likely to seek medical care, this could result in the rates of depression being under-estimated among heavy drinkers.

## Conclusions

In conclusion, low-to-moderate total alcohol intake and specifically wine consumption may reduce incident depression, while heavy drinkers seem to be at higher risk. Further cohort studies are needed to confirm these results.

## Abbreviations

AUD: Alcohol use disorders; CHD: Coronary heart disease; CI: Confidence interval; FFQ: Food Frequency Questionnaire; GEE: Generalized estimating equations; HR: Hazard ratio; MET: Metabolic equivalent task; PREDIMED: (Prevención con Dieta Mediterránea) Prevention with Mediterranean Diet; SUN cohort: University of Navarra follow-up Study (“Seguimiento Universidad de Navarra”).

## Competing interests

Dr. Estruch reports serving on the board of and receiving lecture fees from the Research Foundation on Wine and Nutrition (FIVIN); serving on the boards of the Beer and Health Foundation and the European Foundation for Alcohol Research (ERAB); receiving lecture fees from Cerveceros de España and Sanofi-Aventis; and receiving grant support through his institution from Novartis. Dr. Salas-Salvadó reports serving on the board of and receiving grant support through his institution from the International Nut and Dried Fruit Council; receiving consulting fees from Danone; and receiving grant support through his institution from Eroski and Nestlé. Dr. Arós reports receiving payment for the development of educational presentations from Menarini and AstraZeneca. Dr. Lamuela-Raventós reports serving on the board of and receiving lecture fees from FIVIN; receiving lecture fees from Cerveceros de España; and receiving lecture fees and travel support from PepsiCo. Dr. Serra-Majem reports serving on the boards of the Mediterranean Diet Foundation and the Beer and Health Foundation. Dr. Pintó reports serving on the board of and receiving grant support through his institution from the Residual Risk Reduction Initiative (R3i) Foundation; serving on the board of Omegafort; serving on the board of and receiving payment for the development of educational presentations, as well as grant support through his institution, from Ferrer; receiving consulting fees from Abbott Laboratories; receiving lecture fees, as well as grant support through his institution, from Merck and Roche; receiving lecture fees from Danone and Esteve; receiving payment for the development of educational presentations from Menarini; and receiving grant support through his institution from Sanofi-Aventis, Kowa, Unilever, Boehringer Ingelheim, and Karo Bio. No other potential conflict of interest relevant to this article was reported.

## Authors’ contributions

AG conducted the literature review, participated in the design of the present study, cleaned the data, conducted the main statistical analyses and prepared the first draft of the manuscript. J-JB participated in the statistical analyses plan and in the design of the present study, and revised the manuscript. RE initiated the collaborative project, designed data collection tools, monitored data collection for the whole trial, revised the manuscript and contributed to the interpretation of findings. AS-V conducted part of the literature review, cleaned and analyzed data, participated in the design of the present study, revised the manuscript and contributed to the interpretation of findings. JS-S implemented the trial in Reus, monitored the data collection and revised the manuscript. PB-C participated in the implementation of the trial in Pamplona, participated in the design of the data collection tools and revised the manuscript. EG-G implemented the trial in Málaga, monitored the data collection and revised the manuscript. M-IC implemented the trial in Barcelona, monitored the data collection and revised the manuscript. DC implemented the trial in Valencia, monitored the data collection, and revised the manuscript. MF implemented the trial in Palma de Mallorca, monitored the data collection and revised the manuscript. FA implemented the trial in Vitoria, monitored the data collection and revised the manuscript. JL implemented the trial in Sevilla, monitored the data collection and revised the manuscript. R-ML-R implemented the trial in Barcelona, monitored the data collection and revised the manuscript. JW participated in the design of the present study, monitored the data collection and revised the manuscript. XP implemented the trial in Barcelona, monitored the data collection and revised the manuscript. LS-M implemented the trial in Las Palmas de Gran Canaria, monitored the data collection and revised the manuscript. M-AM-G initiated the collaborative project, designed data collection tools, monitored data collection for the whole trial, implemented the trial in Pamplona, supervised all the steps in the statistical analyses and preparation of the manuscript, and contributed to the interpretation of findings. He is the guarantor. All authors critically revised the manuscript for important intellectual content and approved the final version to be submitted for publication.

## Supplementary Material

Additional file 1 Flow chart of participants: the PREDIMED Study. Click here for file

Additional file 2 **Main analyses stratified by sex.** Hazard ratios (95% confidence intervals) for incident depression according to categories of baseline daily alcohol intake, and Relative risks (95% confidence intervals) of incident depression according to categories of updated alcohol intake, using repeated measurements of diet during follow-up, stratified by sex. The PREDIMED Study 2003 to 2010. Click here for file

Additional file 3 **Fixed-effects models.** Odds ratios (95% confidence intervals) for a new incident episode of depression, or recovery from an episode of depression, according to categories of baseline daily alcohol intake, using fixed-effects models. The PREDIMED Study 2003 to 2010. Click here for file
