# Supplementary figures and images for: Alcohol intake, wine consumption and the development of depression: the PREDIMED study
Source: BMC Med. 2013 Aug 30;11:192. doi: 10.1186/1741-7015-11-192 (PMC3765610; doi:10.1186/1741-7015-11-192)

**Additional File 1.** Flow Chart of Participants: The PREDIMED Study.

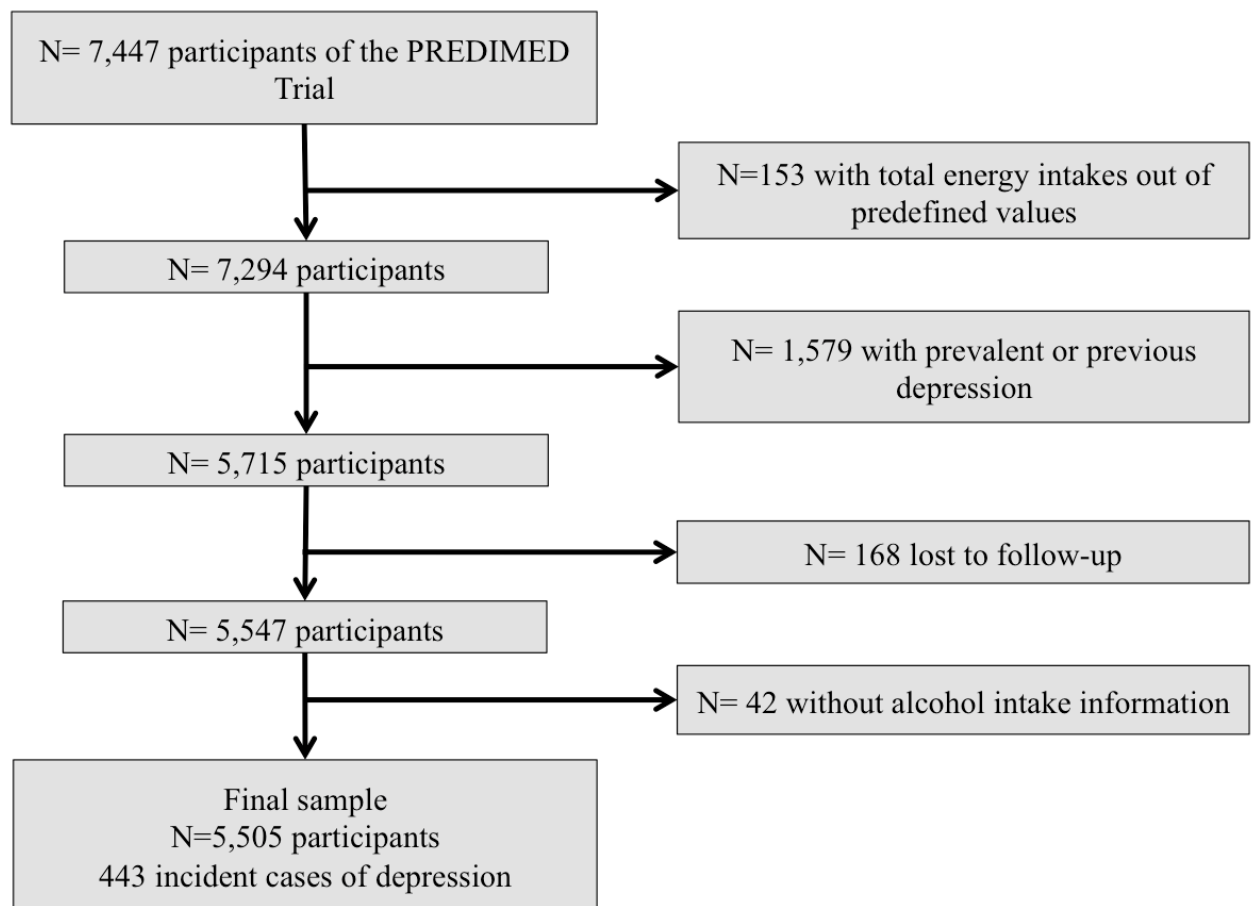

Supplement: Additional file 1 — Flow chart of participants: the PREDIMED Study. [file 1741-7015-11-192-S1.pdf]
