# Supplementary material for: Alcohol intake, wine consumption and the development of depression: the PREDIMED study
Source: BMC Med. 2013 Aug 30;11:192. doi: 10.1186/1741-7015-11-192 (PMC3765610; doi:10.1186/1741-7015-11-192)
Supplement: Additional file 2 — Main analyses stratified by sex. Hazard ratios (95% confidence intervals) for incident depression according to categories of baseline daily alcohol intake, and Relative risks (95% confidence intervals) of incident depression according to categories of updated alcohol intake, using repeated measurements of diet during follow-up, stratified by sex. The PREDIMED Study 2003 to 2010. [file 1741-7015-11-192-S2.pdf]

Additional File 2. Hazard ratios (95% Confidence Intervals) for incident depression according to categories of baseline daily alcohol intake, and Relative risks (95% Confidence Intervals) of incident depression according to categories of updated alcohol intake, using repeated measurements of diet during follow-up, stratified by sex. The PREDIMED Study 2003-2010.

Alcohol intake categories (g/day)	0	>0-5	>5-15	>15	*P* for trend
**MEN**					
	Baseline Alcohol Intake				
Cases/Person-years	22/1,708	26/2,352	28/3,348	45/4,210	
Age-adjusted model	1 (Ref.)	0.86 (0.48-1.53)	0.68 (0.39-1.21)	0.84 (0.50-1.43)	0.868
Multiple-adjusted model[a]	1 (Ref.)	0.84 (0.42-1.66)	0.51 (0.26-1.00)	0.75 (0.39-1.43)	0.909
	Updated Alcohol Intake[b]				
Age-adjusted model	1 (Ref.)	0.60 (0.30-1.23)	0.71 (0.36-1.23)	0.71 (0.37-1.39)	0.677
Multiple-adjusted model[a]	1 (Ref.)	0.60 (0.29-1.23)	0.68 (0.34-1.35)	0.65 (0.33-1.29)	0.828
**WOMEN**					
	Baseline Alcohol Intake				
Cases/Person-years	173/6,069	88/3,376	51/2,042	10/550	
Age-adjusted model	1 (Ref.)	0.92 (0.71-1.19)	0.89 (0.65-1.22)	0.62 (0.33-1.18)	0.169
Multiple-adjusted model[a]	1 (Ref.)	0.99 (0.74-1.31)	0.83 (0.58-1.18)	0.61 (0.30-1.27)	0.192
	Updated Alcohol Intake[b]				
Age-adjusted model	1 (Ref.)	0.76 (0.58-1.00)	0.70 (0.48-1.00)	0.62 (0.31-1.23)	0.216
Multiple-adjusted model[a]	1 (Ref.)	0.77 (0.58-1.01)	0.69 (0.47-1.01)	0.64 (0.32-1.28)	0.275

a Adjusted for age, smoking, physical activity (MET-min/d), total energy intake (Kcal/day), baseline body mass index (kg/m^2), marital status, intervention group, recruiting center, educational level, and number of persons living at home.

b To avoid reverse causality bias, an induction period of at least 1 year, but no longer than 2 years was assumed. We considered as incident cases of depression those occurring only during the second year of every 2-year follow-up interval.
