# Supplementary material for: Alcohol intake, wine consumption and the development of depression: the PREDIMED study
Source: BMC Med. 2013 Aug 30;11:192. doi: 10.1186/1741-7015-11-192 (PMC3765610; doi:10.1186/1741-7015-11-192)
Supplement: Additional file 3 — Fixed-effects models. Odds ratios (95% confidence intervals) for a new incident episode of depression, or recovery from an episode of depression, according to categories of baseline daily alcohol intake, using fixed-effects models. The PREDIMED Study 2003 to 2010. [file 1741-7015-11-192-S3.pdf]

Additional File 3. Odds ratios (95% confidence intervals) for a new incident episode of depression, or recovery from an episode of depression, according to categories of baseline daily alcohol intake, using fixed-effects models. The PREDIMED Study 2003-2010.

Alcohol intake categories (g/day)	0	>0-5	>5-15	>15
Crude model	1 (Ref.)	0.98 (0.83-1.17)	0.92 (0.73-1.16)	0.87 (0.62-1.22)
Age-adjusted model	1 (Ref.)	0.91 (0.66-1.06)	0.84 (0.66-1.06)	0.76 (0.54-1.07)
Multiple-adjusted model[a]	1 (Ref.)	0.91 (0.76-1.08)	0.86 (0.67-1.09)	0.76 (0.53-1.09)

a Adjusted for age, smoking, physical activity (MET-min/d), total energy intake (Kcal/day), baseline body mass index (kg/m^2), marital status, and diabetes.
